# Characterization of partially purified milk‐clotting enzyme from sunflower (*Helianthus annuus*) seeds

**DOI:** 10.1002/fsn3.338

**Published:** 2016-01-15

**Authors:** Assia I. A. M. Nasr, Isam A. Mohamed Ahmed, Omer I. A. Hamid

**Affiliations:** ^1^ Milk Science and Technology DepartmentCollege of Animal Production of Science and TechnologySudan University of Science and TechnologyKhartoum NorthKukuSudan; ^2^ Department of Food Science and TechnologyFaculty of AgricultureUniversity of KhartoumKhartoum NorthShambat13314Sudan

**Keywords:** Enzyme extraction, *Helianthus annuus*, milk‐clotting activity, rennet substitute, Sudanese white soft cheese

## Abstract

This study was aimed to extract milk‐clotting enzyme from sunflower seeds and to determine its potentiality for manufacturing white soft cheese from cows and goats milk. The seeds were blended and extracted using two types of buffers and milk‐clotting and proteolytic activities were evaluated. The enzyme was partially purified using ammonium sulfate fractionation techniques. Results indicated that sunflower seeds extracted with 5% NaCl in 50 mmol/L acetate buffer, pH 5.0, had the highest milk‐clotting activity (MCA) and lowest coagulation time compared to that extracted with only acetate buffer (pH 5.0). Ammonium sulfate at 30–50% saturation purified the enzyme to 4.3 folds with MCA of 241.0 U/mL and final enzyme yield of 10.9%. The partially purified enzyme was characterized by SDS–PAGE that showed two bands with molecular weight of 120 and 62 kDa. When compared with other plant enzymes, the partially purified sunflower enzyme was found to have higher milk‐clotting activity and lower proteolytic activity. Also, both milk sources and enzyme types significantly affected the cheese yield and curd formation time. The cheese made from cow milk using sunflower enzyme had higher yield compared to that obtained using commercial rennet, whereas the opposite was observed when using goat milk.

## Introduction

Milk clotting is the basic step in the manufacture of all types of cheeses and based on that all cheese varieties (>2000 types) are classified into three superfamilies those include rennet coagulated, acid coagulated, and a combination of heat and acid coagulated cheeses (Badgujar and Mahajan 2014; Fox et al. 2015). Rennet coagulated cheese represent the major type (~75%) and calf rennet has been and still the most widely used milk‐clotting enzyme preparation in cheese making industry (Mohamed Ahmed et al. 2009a). However, in the last decades there has been increased demand for cheese production and consumption due to the population explosion (Elsamani et al. 2014; Tajalsir et al. 2014). This combined with the elevated price of calf rennet and reduced quantity of natural calf rennet (Mohamed Ahmed et al. 2010). Together with that, the use of animal rennet is restricted for religious (e.g., Judaism and Islam), safety (bovine spongiform encephalopathy), and diet (vegetarianism) reasons, or being against genetically engineered foods (Badgujar and Mahajan 2014; Roseiro et al. 2003). All the above reasons have demanded the search for a new enzyme with a high ratio of milk‐clotting/proteolytic activity and low preparation cost to be used as a rennet substitute and/or additive (Tajalsir et al. 2014). Consequently, much research interest has been focused on the discovering milk‐clotting enzymes from other sources, and, as a result, several enzyme preparations of animal, microbial, and plant origin have been discovered (Jacob et al. 2011). However, most of the enzyme preparations from the above sources were found unsuitable because they produced cheese with low yield and bitter taste due to the low ratio of milk‐clotting/proteolytic activities (Anusha et al. 2014; Shah et al. 2014). Therefore, the search for a rennet substitute from plant sources having a high ratio of milk‐clotting/proteolytic activity is extremely needed to be used for the production of cheese with better quality.

In Sudan, the seeds of some plants are traditionally used for the production of soft white cheese from goat and sheep milk (Mohamed Ahmed et al. 2009a). These plants sources include *Solanum dubium*,* Solanum innacum*,* Solanum torvaum*,* Solanum esculentum,* and *Solanum melongena*, and many other species (Guiama et al. 2010). Recently, Mohamed Ahmed et al. (2009b) reported the purification and characterization of milk‐clotting serine protease, named as dubiumin, having a high ratio of milk‐clotting/proteolytic activities from the seeds of *S. dubium* and assumed as suitable rennet substitute. Sunflower (*Helianthus annuus* L) is widely distributed in the Sudan, and its seeds are edible and are used for extraction of cooking oil. Previously, the seeds of sunflower were used for the isolation of aspartic protease (Park et al. 2000) with high sequence similarity to cynarase of *Cynara cardunculus* (Cordeiro et al. 1994). However, the sunflower enzyme displays a negligible value of milk‐clotting activity, whereas the cynarase has high milk‐clotting activity (Park et al. 2000). In addition, Egito et al. (2007) studied the milk‐clotting ability of ammonium sulfate precipitated extract of sunflower seeds and investigated its mode of action on different caseins. Despite the aforementioned studies on milk‐clotting enzymes from sunflower seeds, in‐depth evaluation of the potentiality of milk‐clotting enzyme of this plant as rennet substitute still scarce. Therefore, the main aim of this study is isolate milk‐clotting enzyme from sunflower seeds and to investigate its milk‐clotting properties mainly ratio of milk‐clotting activity/proteolytic activity, cheese yield, and curd formation time on different milk sources.

## Materials and Methods

### Materials

Fresh whole cow milk (50 L) was brought from College of Animal Production Science and Technology farm, Sudan University of Science and Technology, Hillat Kuku, Khartoum, Sudan. Fresh Saanen goats' milk was brought from a local farm at Hillat Kuku, Khartoum, Sudan. Rennet powder was obtained from Chr. Hansen's lab, Denmark. Clean and fine NaCl was purchased from local market. Each milk was filtered and divided into two equal volumes of (25 L) each and kept 4°C in separate containers until used for cheese making. The decorticated sunflower seeds were obtained from Abo‐Ras factory for sunflower seeds' products, Khartoum, Sudan. The seeds of sunflower were carefully cleaned, and then coarsely grounded using an electric grinder and kept in polyethylene bags at 4°C until used for enzyme extraction.

### Enzyme extraction and purification

The crude extract of sunflower seeds enzyme was prepared using different extracting buffers as described previously (Mohamed Ahmed et al. 2009a). Briefly, duplicate samples of 10 g of sunflower seeds powder were immersed in 100 mL of sodium acetate buffer with or without 5% NaCl (w/v) with continuous stirring. The extraction procedure was continued for 24 h at 4°C. The extracts were filtrated through cheese cloth and centrifuged at 5000 × g for 20 min. The supernatant was used for enzyme purification by ammonium sulfate fractionation method. Initially, solid ammonium sulfate was slowly added with stirring to the crude extract (600 mL) preparations to 30% saturation. The pH of the enzyme/ammonium sulfate solution was maintained at pH 5.0 by a dropwise addition of either 0.1 mol/L H_2_SO_4_ or 7% NH_4_OH, and the mixture was then maintained on ice for 30 min. Thereafter, the precipitates were separated from the supernatant by centrifugation at 8000 ×g (Centurion Scientific Co., Ltd, west Sussex, UK) for 20 min at 4°C. Solid ammonium sulfate was further slowly added to the supernatant to 55% saturation, and the solution was preserved on ice for another 30 min before being centrifuged at 8000 ×g for 20 min at 4°C. Solid ammonium sulfate was again further slowly added to the supernatant to 80% saturation with ammonium sulfate, and then the solution was kept on ice for another 30 min before being centrifuged at 8000 ×g for 20 min at 4°C. The precipitates collected were dissolved in a small volume (about 2 mL) of 50 mmol/L Na acetate buffer, pH 5.0, and then dialyzed overnight at 4°C against the same buffer (10 L). The extract was then centrifuged, and the supernatants were examined for milk‐clotting and protease activities as well as protein concentration. The partially purified enzyme was either directly used for enzyme characterization and cheese making or freeze‐dried at −50°C. The latter step was repeated until the quantity of the freeze‐dried partially purified enzyme reached 2 g and stored at −20°C until used for cheese making and enzyme characterization.

### Determination of milk‐clotting activity

The milk‐clotting activity was determined following the procedure described by IDF (1992). Briefly, 60 g of skimmed milk powder (Confectionery skim milk powder, Poland) was reconstituted in 500 mL of 0.01 mol/L CaCl_2_ (pH 6.5) and the mixture was stored at 4°C. The extract was added at a proportion of 0.2 mL per 1.0 mL of milk (0.2:1.0, v/v). The clotting point was estimated during the manual shaking of the test tube, at very short time intervals. The coagulation time was documented when separate particles were noticeable. One milk‐coagulating unit (U) was defined as the amount of the enzyme that coagulates 10 mL of reconstituted skimmed milk powder at 30°C in 100 sec (Berridge 1952).

### Determination of protease activity

The protease activity was determined following the method described by Sarath et al. (1989) using casein as substrate.

### Determination of the protein contents

The protein content of the crude extract and fractions obtained after the partial purification of the enzyme was estimated by measuring the extinction at 280 nm. Quantitative determination of the protein content was estimated according to Bradford (1976) method. Briefly, 2.5 mL of Bradford reagent was added to 0.25 mL of the samples and BSA (Bovine serum albumin) solution as standard (both at different dilutions) and incubated for 10 min, and then the absorbance was read at 595 nm. The protein content was calculated from the BSA standard curve.

### SDS‐PAGE and zymography of sunflower enzyme

The lyophilized samples (5 mg) were reconstituted in 1 mL of 125 mMTris‐HCl buffer pH 6.8 containing 2.5% SDS, 1% sucrose, and 0.05% bromophenol blue (Egito et al. 2007). Then, 25 *μ*L (60 *μ*g) protein was loaded onto SDS‐PAGE containing 0.1% gelatin or casein. The gel without substrate was used for SDS‐PAGE analysis. The electrophoresis was carried out using 4% stacking gel and 12% resolving gel at 20 mA/gel for 90 min at 4°C. After the electrophoresis, the gels were either directly stained with CBB for 1 h at room temperature (SDS‐PAGE) or washed twice with the renaturing solution (100 mL of 2.5% of Triton X‐100 containing 15 mmol/L CaCl_2_) for 30 min (Zymography). The gel was then washed several times with distilled water to remove the renaturing solution. The hydrolysis reaction was then proceeded inside the gel by the incubation of the gel in the developing solution (50 mmol/L Tris‐HCl buffer, pH 7.5, containing 15 mmol/L CaCl_2_) at 37°C for 30 h. Thereafter, the gel was washed twice with distilled water and immediately stained with CBB solution (0.5% Coomassie blue R‐250, 5% methanol, and 10% acetic acid in distilled water) for 1 h at room temperature. Then, the gel was destained with destaining solution of 10% methanol and 5% acetic acid in dH_2_O for several hours. The active bands were appeared as translucent bands on the blue background.

### Cheese making

The cheese was produced according to the procedure of Talib et al. (2006, 2009) with slight modifications. Briefly, 25 L of fresh cow and goats' milk was heated at 72°C for 15 sec and then cooled to 45°C, and CaCl_2_ was added at the rate of 0.02%. Then, a starter culture of lactic acid (*Lactobacillus bulgaricus* and *Lactobacillus thermophilus*) was added at the rate of 2.0% w/v and left for 30 min to develop acidity. Rennet tablets (one tablet/45 kg milk) for control ones and the partially purified freeze‐dried enzymes of the sunflower seeds were added to the milk at the rate of 2 g/50 L of milk. The milk was mixed and left until coagulation completed. Rennet coagulation times were recorded for each treatment. After coagulation, the curd was cut vertically and horizontally into 5 cm^3^ with a sharp knife accompanied by the addition of 3% NaCl (w/v) for each treatment. The whey obtained from the cheese curd was drained, and the curd was poured into small wooden boxes lined with cloth and pressed overnight. The curd was removed from the wooden boxes and cut into cubes of 10 × 5 × 5 cm^3^, then the cheese yield was recorded for each treatment. The percentage of cheese yield was calculated as follow:

$$\mathrm{Cheese}\;\mathrm{yield}\;(\%)\;=\;\frac{\mathrm{Weight}\;\mathrm{of}\;\mathrm{cheese}\;(\mathrm{kg})}{\mathrm{Weight}\;\mathrm{of}\;\mathrm{milk}\;(\mathrm{kg})}\;\times \;100$$

### Statistical analysis

The data of three independent experiments were collected and statistically analyzed using analysis of variance (ANOVA) and Duncan's multiple range test (DMRT). Significance was accepted at *P* ≤ 0.05, and *P* ≤ 0.01.

## Results

### Extraction and partial purification of milk‐clotting enzyme

The preliminary experiments (Table 1) showed that sunflower seeds extracted with 5% NaCl in 50 mmol/L sodium acetate buffer, pH 5.0, had significantly (*P* ≤ 0.05) higher milk‐clotting activity compared to that extracted with the same buffer without adding 5% NaCl. In addition, a significant reduction in clotting time was observed when using 5% NaCl in sodium acetate buffer. Thus, 5% NaCl in sodium acetate buffer, pH 5.0 was selected as an extracting buffer and was used throughout the study. The extraction period for the enzyme was varied from 6 to 120 h, and it was noted that the extraction time had a significant (*P* ≤ 0.01) effect on the clotting time, and best results were shown in 24 h (Table 2). Thus, this extraction time was used throughout the study.

**Table 1:** Effect of extractant on milk‐clotting activity and coagulation time of enzyme extract from *Helianthus annuus* seeds

Extractant	Milk‐clotting activity (U/mL)	Clotting time (sec)
50 mmol/L Na acetate buffer (pH 5.0)	160.0 ± 4.59^b^	26.00 ± 3.16^a^
5%NaCl in 50 mmol/L Na acetate buffer (pH 5.0)	185.3 ± 4.50^a^	21.13 ± 3.38^b^
Significance level	a	a

Mean values (M ± SD) bearing different superscripts within the same column are significantly different (*P* ≤ 0.05).

a Significant (*P* ≤ 0.05).

**Table 2:** The effect of extraction time on the clotting time

Extraction time (h)	Clotting time (sec)	Significance level
6	30.00 ± 2.94^ab^	a
12	20.00 ± 1.63^bcefg^	a
24	13.50 ± 3.11^cdfgh^	a
36	21.00 ± 5.89^bcdef^	a
48	22.00 ± 5.89^bcde^	a
72	23.50 ± 5.32^bcd^	a
96	26.00 ± 3.65^abc^	a
120	32.50 ± 1.91^a^	a

Mean values (M ± SD) bearing different superscripts within rows are significantly different (*P* ≤ 0.05).

a Significant (*P* ≤ 0.01).

Salting out using ammonium sulfate precipitation was performed as the sole step for the purification of sunflower milk‐clotting enzyme. The results showed that the 30‐50% ammonium sulfate saturation fraction had the highest milk‐clotting activity compared to other fractions (data not shown). The concentration at this range was used for partial purification of the enzyme. The simple purification procedure developed in this study, applying only ammonium sulfate, resulted in 4.3 folds purity with a yield of 10.87% and specific activity of 0.29 U/mg protein (Table 3).

**Table 3:** Summary of the partial purification of milk‐clotting enzyme from sunflower enzyme seeds

Purification steps	Volume (mL)	Milk‐clotting activity (units/mL)	Protein content (mg/mL)	Total activity (units)	Specific activity (units/mg)	Yield (%)	Purification (fold)
Crude extract	30.0	185.3	2767.0	5559.0	0.07	100.0	1.0
AmSO_4_ (30–50%)	2.5	241.0	837.0	604.0	0.29	10.9	4.3

### Ratio of milk clotting to proteolytic activity of the purified enzyme

Table 4 summarizes the milk‐clotting and proteolytic activities of the partially purified enzyme compared to other coagulants. Sunflower enzyme had higher clotting and lower proteolytic activities compared to other plant enzymes. It was found that clotting and proteolytic activities of the partially purified enzyme were 241.6 units/ml and 0.07 (OD 660 nm), respectively, and that of rennet were 249.6 units/mL and 0.05 (OD 660 nm), respectively. Strikingly, the ratio of milk clotting to the proteolytic activity of sunflower enzyme (3451.4) was comparable to those of commercial rennets mainly Calf (4992.0) and Mucor (4650.0) rennet. This result indicates the potentiality of sunflower milk‐clotting enzyme to be used in cheese making as rennet substitute or additive.

**Table 4:** Ratio of milk‐clotting activity/proteolytic activity of sunflower seeds enzyme and other coagulants

Enzyme	Clotting activity (units/mL)	Proteolytic activity (OD 660 nm)	Ratio (units/OD 660 nm)
Rennet	249.6	0.05	4992.0
Mucor rennet	551.0	0.11	4650.0
Sunflower enzyme	241.6	0.07	3451.4
Dubiumin	880.0	0.35	2490.0
Papain	216.0	0.59	367.0

### Polyacrylamide gel electrophoresis (SDS‐PAGE and Zymography)

**Figure 1:**
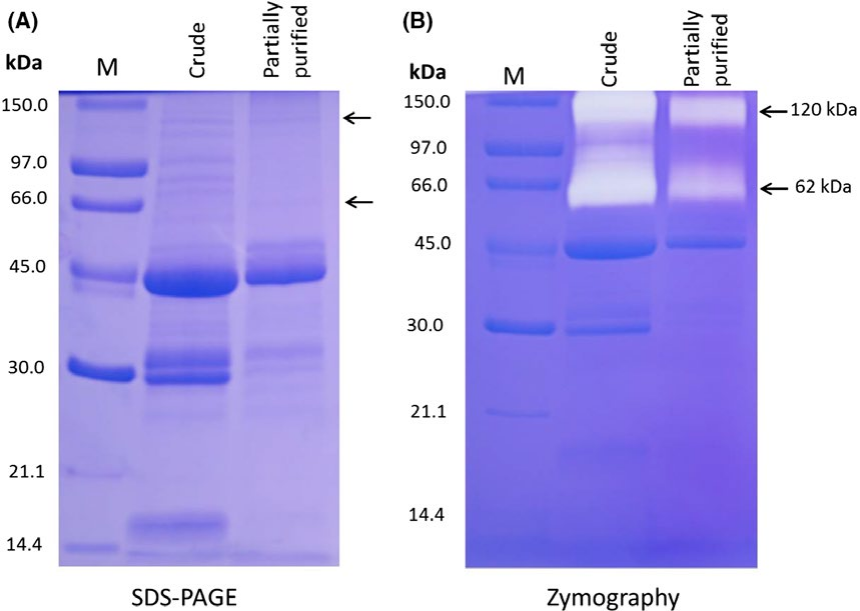
(A) SDS‐PAGE, and (B) activity staining (Zymography) of partial purified milk‐clotting enzyme from *Helianthus annuus* seeds. In both, Lane 1 molecular weight standards from high molecular weight descending; MBP‐*β*‐ galactosidase, Phosphorylase b, Bovine Serum Albumin, Ovalbumin, Carbonic anhydrase, Soybean trypsin inhibitor, and *α*‐Lactalbumin. Lane 2, (60 *μ*g) crude extract of sunflower. Lane 3 (60 *μ*g) active partially purified sunflower enzyme.

**Figure 2:**
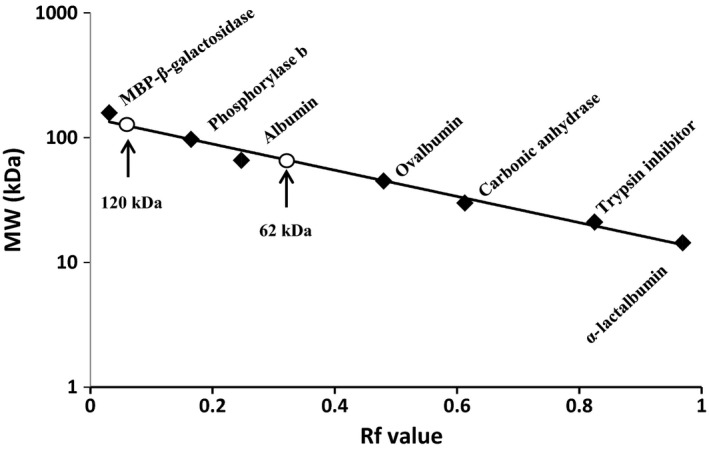
Estimation of the molecular mass of sunflower seeds milk‐clotting enzyme by SDS‐PAGE. Semi‐logarithmic plot of relative molecular masses versus their relative mobility (Rf value).

Polyacrylamide gel electrophoresis under reducing conditions (SDS‐PAGE) was used to check the purity, activity, and to determine the molecular mass of partially purified enzyme. The SDS‐PAGE gel showed several bands of both crude extract and partially purified enzyme (Fig. 1A) indicating that there are still contaminating protein not yet removed. However, the activity staining using gelatin as substrate showed two active bands with high‐molecular weight (Fig. 1B). The molecular mass of the enzyme was determined by comparison of the migration distance of the protease to that of standard marker proteins. The molecular mass of the partially purified enzyme was calculated to be 120 and 62 kDa (Fig. 2), which coincided with the bands of MBP‐*β*‐galactosidase and bovine serum albumin marker proteins.

### Cheese processing and analysis

**Figure 3:**
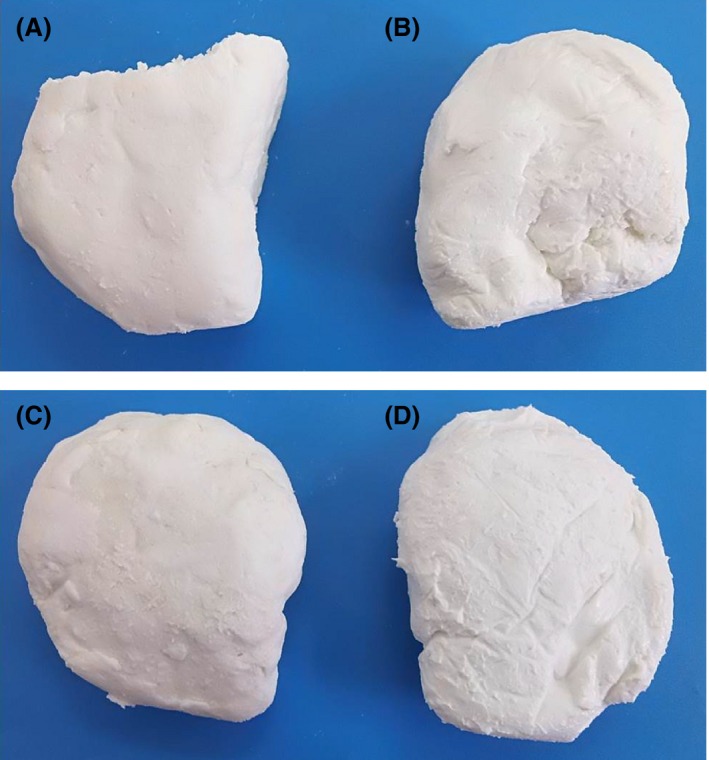
Cheese produced using different milk types and enzyme source. The upper photos showed cow milk cheese processed using calf rennet (A) and the partially purified enzyme of sunflower seeds (B). While the lower photos showed, goat milk cheese produced using calf rennet (C) and the partially purified enzyme of sunflower seeds (D).

In order to evaluate the potentiality of the partially purified milk‐clotting enzyme of sunflower seeds in cheese making soft white cheeses was made from cow and goat milks. These cheeses were evaluated for their yield and coagulation time as well as curd formation and were compared with those of commercial calf rennet. Regardless of the milk type used, the enzyme greatly coagulated fresh milk and formed a white and soft cheese (Fig. 3B and D) compared to that of calf rennet (Fig. 3A and C). This slight variation in cheese hardness of both enzymes was supported by the results of cheese yields (Table 5). The results showed a significant difference (*P* ≤ 0.01) in the yield percentage of cheese made from cow milk compared to that of goat milk. However, there is no significant difference in the coagulation time between the two kinds of milk. The effect of coagulant type on the yield percentage and coagulation time of soft white cheese is presented in Table 6. The results revealed a significant (*P* ≤ 0.05) difference in the yield percentage of cheese produced by rennet and sunflower extract. The results of sunflower cows cheese had higher yield percentage compared to those from rennet. On the other hand, the results showed high significant (*P* ≤ 0.01) differences in coagulation time of rennet and sunflower enzyme. Table 7 showed the effect of interaction between milk source and coagulant type on the yield percentage and coagulation time of the manufactured white soft cheese. The results showed high significant (*P* ≤ 0.01) differences in the interaction between milk source and coagulant type on the yield percentage. Whereas, no significant differences were noted for the interaction of milk source and coagulant type on coagulation time.

**Table 5:** Effect of milk source on the yield and coagulation time of soft white cheese

Parameters	Milk source		Significance level
	Cow	Goat	
Yield (%)	21.60 ± 3.43^a^	17.90 ± 2.03^b^	a
Coagulation time (h)	2.21 ± 1.45	2.31 ± 1.67	NS

Mean values (M ± SD, *n* = 3) bearing different superscripts within rows are significantly different (*P* ≤ 0.01). NS, not significant.

a Significant (*P* ≤ 0.01).

**Table 6:** Effect of coagulant type on the yield and coagulation time of soft white cheese

Parameters	Coagulant type		Significance level
	Rennet	Sunflower enzyme	
Yield (%)	18.73 ± 1.10^b^	20.78 ± 5.37^a^	*
Coagulation time (h)	0.91 ± 0.07^b^	3.60 ± 0.27^a^	**

Mean values (M ± SD, *n* = 3) bearing different superscripts within rows are significantly different (*P* ≤ 0.05).

*Significant (*P* ≤ 0.05). **Significant (*P* ≤ 0.01).

**Table 7:** Effect of milk source and coagulant type on the yield and coagulation time of the manufactured white soft cheese

Parameters	Milk source				Significance level
	Cow		Goat		
	Rennet	Sunflower enzyme	Rennet	Sunflower enzyme	
Yield (%)	17.80 ± 0.39^b^	25.40 ± 0.39^a^	19.65 ± 0.35^a^	16.15 ± 0.07^b^	a
Coagulation time (h)	0.95 ± 0.07	3.47 ± 0.02	0.88 ± 0.06	3.74 ± 0.37	NS

Mean values (M ± SD, *n* = 3) bearing different superscripts within rows are significantly different (*P* ≤ 0.05).

a Significant (*P* ≤ 0.01).

## Discussion

Globally, cheese is one of the oldest and diverse dairy products manufactured about 8000 years ago and having more than 2000 types (Fox et al. 2015). This huge diversity in cheese type resulted mainly from the coagulation method, which considered as the crucial step in cheese making. Coagulation using rennet‐like enzymes is the major procedure representing about 75% of cheese production (Fox et al. 2015). However, the increased demand for cheese as a convenience food, dessert or snack, a sandwich filler, and food ingredient, has resulted in increased requirements for milk coagulants with good coagulation properties and cheap production cost. Thus, the search for the milk‐clotting enzyme from various sources has been on the rise, and accordingly in this study, the seeds of sunflower were used to partially purify milk‐clotting enzyme. Various extracting systems were applied, in terms of extracting buffers and time, and found that using 5% NaCl in 50 mmol/L Na acetate buffer, pH 5.0, for 24 h was effective in extracting the milk‐clotting enzyme from sunflower seeds. Our results are in agreement with the observation of Mohamed Ahmed et al. (2009a) and Talib et al. (2009) who reported that buffer extract of *S. dubium* berries had less milk‐clotting activity than extract with 5% NaCl sodium acetate buffer. An increase in the buffer salts strength of the extracting solution increases the milk‐clotting enzyme, and, therefore, allows the extraction increasing. In addition, by using a simple purification procedure (ammonium sulfate precipitation), some of the total protease in the crude extract was removed out. This simple purification procedure not only resulted in the effective removal of the partial proteases and colored materials which existing in the crude extract (Barros et al. 2001) but it also concentrated the enzyme preparation to a workable volume that could be used for enzyme characterization and cheese making. The partial purification procedure developed in this study resulted in two active bands with a molecular mass of 120 and 62 kDa as judged by SDS‐PAGE and zymogram activity staining. SDS‐PAGE gel still showing other contaminating proteins revealing the needs for further purification steps. However, this will increase the production cost of the enzyme. Thus, partial purification of the enzyme using ammonium sulfate is recommended as it led to clear enzyme preparations with excellent milk‐clotting properties and cheese making potential. The cheese making potential of the partially purified milk‐clotting enzyme from sunflower seeds was apparent from its high ratio of milk‐clotting/proteolytic activity as well as the firm curd formation. The ratio of milk clotting to proteolytic activity is a valuable indicator of the protease efficiency to be used as a coagulant for cheese manufacture (Arima et al. 1970). The presence of two active bands of the partially purified enzyme might be an indicator of either a heterodimer protease with two enzyme subunits or of two different enzymes. The latter assumption could explain the two bands in this study as the molecular masses of these bands are comparable to those reported for other milk‐clotting enzymes. For instance, the molecular weight of the 62 kDa band of the partially purified milk‐clotting enzyme is similar other known milk‐clotting enzymes of plant sources, (Kumari et al. 2012; Mohamed Ahmed et al. 2009b). Whereas, the other band of 120 kDa molecular weight is close to that reported by Egito et al. (2007) for milk‐clotting enzyme of *Helianthus annuus* seeds, however; it slightly disagreed with him in resulting in two bands instead of one band of molecular weight 110 kDa. This difference might be due to the difference in the purification procedures between the two studies. Moreover, the zymogram activity staining also showed two clear zones of proteolytic activity against a blue background. Although the sample in the zymogram gel was treated with 2.5% SDS, the enzyme still displayed activity, indicating that the partially purified milk‐clotting enzyme of sunflower seeds is resistant to SDS denaturing, and similar observation has been reported previously (Egito et al. 2007). This property might pave the way of the partially purified milk‐clotting enzyme of sunflower seeds to be used in other biotechnological applications under harsh conditions, besides its potential uses in cheese making industry.

In order to further confirm the suitability of the partially purified enzyme as rennet substitute in cheese making, the enzyme was used for the production of cheese from two types of milk (cow and goats). The produced cheese was evaluated for the yield and curd formation time in comparison with that of commercial calf rennet. These information are crucial to evaluate the potentiality of the purified enzyme from both industrial and commercial standpoints. The findings demonstrated high significant differences in the percentage yield of cow's milk cheese compared to goats' one with no significant differences in the coagulation time using both kinds of milk. The differences in cheese yield between the two types of milk might be due to the difference in the biochemical composition mainly fats, proteins and total solids of both kinds of milk. Cow milk has higher total solids and protein than goat milk, whereas the later milk has higher fats compared to cow milk (Fox et al. 2015). This might explain the higher yield of cheese made from cow milk compared to that made from goat milk. On the other hand, the coagulant type also affects the cheese yield, and the results showed a significant difference in the yield percentage of cheese produced by commercial calf rennet and partially purified sunflower milk‐clotting enzyme. The results of sunflower cows' cheese had higher yield percentage compared to those from rennet; this might be attributed to the fact that the yield of cheese increased due to the incorporation of whey proteins (Abdel Razig 1996; Zaki et al. 1974; Ustunnol and Brown 1985). This could be explained by higher retention of water and the softer cheese formed by sunflower enzyme compared to that formed by rennet. In addition, Abdul‐Rahaman (2013) stated that high content of moisture in cheese directly affect the yield percentage, since moisture considers one of the fundamental factors influencing the increase or decrease in yield percentage. In contrast, our results were disagreed with the results of Abdul‐Rahaman (2013) who concluded that the yield of cheese made using safflower (12.73%) enzyme extract was lower than that made using calf rennet (13.8%). This divergence could be attributed to the using of crude extracts compared to our partial purified enzyme in cheese processing besides the variation in the seeds extracts between sunflower and safflower, the clotting efficiency and the source of milk and variation in its composition (El‐ghazali 2011). On the other hand, the results showed high significant differences in coagulation time of rennet and sunflower enzymes. This findings were in accordance with those of Talib et al. (2009), El‐Owni et al. (2011) and Abdul‐Rahaman (2013) who stated that cheese processed from vegetable extract had higher coagulation time compared to rennet ones. This might be attributed to the using of crude and partially purified extracts in cheese processing compared to commercial rennet.

## Conclusion

We develop a simple purification procedure for the production of substantial amounts of active milk‐clotting enzymes from sunflower seeds as a cheap milk‐clotting preparation for cheese making. In addition, the partially purified enzyme demonstrated great milk‐coagulation specificity and curd formation ability comparable to that of commercial rennet indicating its potentiality in cheese making industry as rennet substitute or additive. However, the further study shall specifically focus on the complete purification of this enzyme, together with in‐depth biochemical, microbiological and sensory evaluation of the produced cheese.

## Conflict of Interest

The authors declare to have no conflict of interest.
